# Structure, Reliability, and Validity of the Bengali Version of the Pain Catastrophizing Scale in Musculoskeletal Chronic Pain Patients in India

**DOI:** 10.7759/cureus.109672

**Published:** 2026-05-26

**Authors:** Soumyajyoti Ghoshal, Santi Ranjan Dasgupta, Subhadip Paul, Arkadeb Dutta

**Affiliations:** 1 Department of Biomedical Science and Technology, Ramakrishna Mission Vivekananda Educational and Research Institute (RKMVERI), Kolkata, IND; 2 Department of Orthopaedics and Pain Management, ESI (Employees' State Insurance) Institute of Pain Management, Kolkata, IND; 3 Department of Sports Science and Yoga, Ramakrishna Mission Vivekananda Educational and Research Institute (RKMVERI), Kolkata, IND

**Keywords:** bengali version, chronic pain, cultural adaptation, factor analysis, pain catastrophizing scale, reliability, validity

## Abstract

Background: Elevated pain catastrophizing increases the severity of pain experience. The 13-item Pain Catastrophizing Scale (PCS) can aid in managing chronic pain. Chronic musculoskeletal pain (CMSP) is common among the native Bengali population in India seeking treatment.

Purpose: This study aimed to culturally adapt and psychometrically validate the original PCS under the Consensus-based Standards for the Selection of Health Measurement Instruments (COSMIN) guidelines, with a novel goal to examine the factor structure of pain catastrophizing in native Bengali CMSP patients in India and compare it with the theoretical pain appraisal model.

Study objectives: The primary objective of this study was to translate and cross-culturally adapt the original PCS into a Bengali version for use in India (BNGA-IND-PCS). The secondary objective was to examine the psychometric properties of BNGA-IND-PCS in patients with CMSP.

Methods: The Bengali version of the PCS for India (BNGA-IND-PCS) was created through forward and backward translation, evaluated by a committee of clinical and language experts, and pre-tested in 30 CMSP patients. The sample size was determined from a 10:1 sample-to-item ratio. A total of 253 CMSP patients (pain duration ≥ 6 months) from convenience sampling were included in the cross-sectional observational study to perform psychometric validation, comprising factor structure determination, internal consistency, test-retest reliability, and concurrent validity (pain severity, anxiety, and depression).

Results: Confirmatory factor analysis showed that the BNGA-IND-PCS two-factor model (pain reflection and helplessness) provided the best fit with a borderline acceptable root mean square error of approximation of 0.06. Internal consistency (Cronbach’s α = 0.921) and test-retest reliability (intra-class correlation coefficient = 0.852, 95% CI: 0.772-0.903) of total BNGA-IND-PCS scores were good to excellent and adhered to the COSMIN criteria. Total BNGA-IND-PCS scores moderately correlated with pain severity, anxiety, and depression (r = 0.406-0.549, p < 0.01).

Conclusion: The PCS translation and cross-cultural adaptation into Bengali demonstrated a two-factor structure and good-to-excellent psychometric properties in a single-sample validation. Future studies with large-scale patient samples are required to test the "ecological validity" under clinical settings.

## Introduction

Chronic pain is a significant health issue with an estimated global prevalence between 9.9% and 50.3% [1]. Chronic pain is common in musculoskeletal conditions, such as low back pain [2], arthritis [3], and fibromyalgia [4], affecting daily life routine. Physical disability and emotional distress (anxiety, depression) in chronic musculoskeletal pain are often associated with misinterpretation of actual pain experience [5-7]. The misinterpretation is primarily psychological [7,8] with a tendency to exaggerate the perceived threat value of anticipated pain experiences [8]. This psychological component, termed pain catastrophizing (PC), can dictate an individual’s evaluation of pain-oriented threat and coping that builds the theoretical pain appraisal model [8]. Hence, a higher level of PC can disrupt standard pain processing and intensify pain experiences [8-10].

The 13-item Pain Catastrophizing Scale (PCS), rated on a five-point Likert scale ("not at all" rated as 0 to "all the time" rated as 4), was originally developed in English [7] to assess PC [11-13]. The original PCS consists of three subscales: helplessness (items 1-5 and 12), magnification (items 6, 7, and 13), and rumination (items 8-11). Helplessness reflects feelings of failure to cope with pain, while magnification and rumination relate to the negative appraisal and focus on pain, respectively. The total PCS score can reach a maximum of 52, with subscales for helplessness, magnification, and rumination having maximum scores of 24, 12, and 16, respectively.

To assess the level of PC, most investigators developed reliable, cross-culturally adapted versions of the PCS across countries and diverse populations. They found that the level of catastrophizing and its subscales (factors) tend to vary across cultures and ethnic groups [14-19]. This underscores the importance of administering language- and culture-oriented, conceptually equivalent PCS to avoid potential misinterpretations of outcomes and inaccurate estimates of related constructs [20]. These adapted versions exhibited good-to-excellent psychometric properties, including internal consistency, test-retest reliability, and validity, suggesting that the PCS is reliable for assessing PC across cultures. Most of these validated versions are based on the traditional three-factor theoretical pain appraisal model, and several times on a two-factor solution [8,21-25].

Earlier, the PCS has been used for chronic musculoskeletal pain conditions, such as low back pain [18,20,26,27] and fibromyalgia [28]. In India, older adults experience debilitating joint and back pain, and the pain experience varies significantly across Indian provinces [21,29]. The prevalence is higher among females, economically disadvantaged individuals, those with lower education levels, and rural populations. The occurrence of back and joint pain is 20.94% and 26.78%, respectively, among approximately 97.2 million native Bengali speakers in India [21]. There exists limited evidence on PC in the Indian population and how it drives pain experience in musculoskeletal disorders. Previously, a single-center study examined PC in 100 Indian patients with chronic low back pain using the Hindi version of the PCS [20]. However, administering the Hindi PCS version across India is difficult due to linguistic and cultural diversity. Hence, rigorous linguistic and cultural adaptation in other spoken Indian languages, tailored to each specific population and guided by established frameworks for validating patient-reported outcomes across cultures, is crucial [20]. The present study attempted to cross-culturally adapt a Bengali version of the original PCS (BNGA-IND-PCS) to understand the psychological factors influencing pain experience in non-malignant musculoskeletal illnesses in India’s second-largest linguistic community [30]. Earlier, a Bengali version of the original PCS was translated and adapted for Bengali-speaking natives in Bangladesh [31]. However, the study did not validate the theoretical pain appraisal model using exploratory and confirmatory factor analysis. This step is crucial to avoid any potential conflict in validating PC factors, either with the traditional three-factor (helplessness, rumination, magnification) pain appraisal model or the occasional two-factor (helplessness, pain focus) structure during cross-cultural adaptation [31]. Hence, we wanted to fill the gap in the literature by validating pain-catastrophizing thoughts in the native Bengali population in accordance with either of the pain appraisal models found across cultures [9]. The Bangladesh PCS version reported that their single-center study of 90 patients in a tertiary care setting might not represent the entire Bengali population of the Indian subcontinent [31]. Despite the standard Bengali language in India and Bangladesh having similar roots, colloquially used Bengali in India has a Sanskrit-derived vocabulary, in contrast to the Persian-Arabic lexicons used in Bangladesh due to cultural influence [32,33]. Therefore, we emphasized the translation of a culturally oriented, conceptually appropriate Bengali version of the original PCS for the native Bengali population in India to avoid possible misinterpretations of PC-related thoughts [34].

Thus, the primary objective of this study was to translate and cross-culturally adapt the original English version of the PCS into BNGA-IND-PCS. The secondary objective was to conduct a psychometric validation analysis of BNGA-IND-PCS. We tested factor structure, internal consistency, test-retest reliability, and concurrent validity as part of the psychometric validation. A cross-sectional observational design was chosen to ensure broad participation of the Indian Bengali population with diverse backgrounds at a single time point. We hypothesized that the BNGA-IND-PCS would demonstrate a two- or three-factor structure, good-to-excellent internal consistency, excellent three-week test-retest reliability, and moderate-to-strong concurrent validity with pain intensity, anxiety, and depression, in accordance with the Consensus-based Standards for the Selection of Health Measurement Instruments (COSMIN) [9,35].

## Materials and methods

Translation and cultural adaptation of the original PCS were performed with permission from the PCS copyright owner, Mapi Research Trust [34]. The linguistic validation study followed recognized guidelines for translating patient-reported outcome measures [36]. The subsequent step involved assessing the psychometric properties of the BNGA-IND-PCS.

Study design

**Figure 1:**
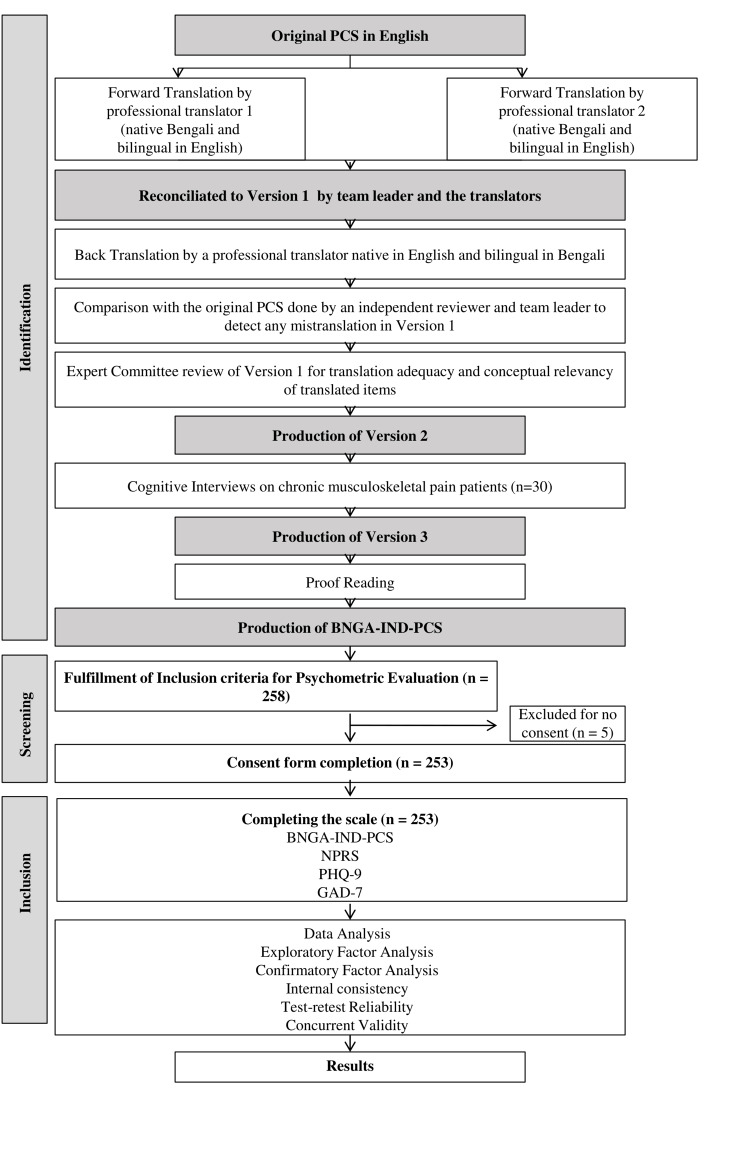
Flow diagram of the linguistic validation and cross-cultural adaptation of the BNGA-IND-PCS. PCS: Pain Catastrophizing Scale; BNGA-IND-PCS: Bengali translation of the Pain Catastrophizing Scale developed in India; NPRS: Numerical Pain Rating Scale; PHQ-9: Patient Health Questionnaire; GAD-7: Generalized Anxiety Disorder-7.

The cross-sectional observational study was conducted from January 2024 to November 2024 and recruited patients through convenience sampling from the Outpatient Department of ESI Hospital, KCM Diagnostic Center, in Kolkata, and from two rural health check-up camps in West Bengal, India. The study included the treatment-seeking population. The inclusion criteria were male and female native Bengali patients aged 25 years or older with chronic musculoskeletal pain lasting at least six months. The diverse musculoskeletal conditions (low back pain, osteoarthritis, rheumatoid arthritis, fibromyalgia-adjacent musculoskeletal disorder, etc.) were confirmed strictly through consistent diagnosis and clinical examination by the medical doctors. Exclusion criteria included visual impairment, inability to understand instructions, psychological disorders, and any other painful conditions. Local announcements and flyers distributed through community liaisons were used to organize the rural camps. The registered patients of the camps were informed about the purpose of the research. Basic demographics were recorded, followed by clinical evaluation. After clinical care, the research assistant received the patient and explained the study. With the patient’s consent to participate, they were screened according to the inclusion and exclusion criteria and enrolled in a self-administered study conducted in an acoustically isolated room. Of 258 eligible patients, 253 native Bengali speakers (male: 100; female: 153; mean age: 54.7 years) completed the study. The reasons for exclusion are shown in Figure 1. The minimal literacy level of the patients was sufficient to self-administer the BNGA-IND-PCS while focusing on their ongoing pain without difficulty. The patients were given both verbal and written instructions about the purpose, procedure, and benefits of the investigation. Written informed consent was obtained from all patients. The study protocol adhered to the Declaration of Helsinki and was approved by the Institutional Ethics Committee (approval number: RKMVERI/IEC/DSSY/2022-11/1).

Sample size calculation

Based on a 10:1 sample-to-item ratio [37], the minimum sample size required for the 13-item PCS was 130. An a priori power analysis was also conducted using G*Power 3.1.9.7 (Heinrich Heine University Düsseldorf, Düsseldorf, Germany) [22] to estimate the minimum sample size. Power analysis determines the minimum sample size by considering the part of the model with the largest number of predictors [38]. The study employed a fixed-effects multiple linear regression model with an effect size of 0.15 to assess the association, using R² as a measure of explained variance. The model included three predictors, with a target power of 90% at a 0.05 level. The minimum sample size was estimated at 99. This multicenter study included 253 patients, exceeding the minimum sample size requirement.

Translation and cross-cultural adaptation of the PCS

The linguistic validation process began with forward translation, followed by backward translation, expert review, and cognitive interviews [36]. The project leader recruited two local, professional, bilingual translators (Bengali and English). These translators independently translated the content and, under the project leader's supervision, reconciled differences to produce the first Bengali version. A third professional translator, native in English and bilingual in Bengali, performed the back-translation of the first version. The back translator was unfamiliar with PCS and was kept blind to the original instrument. A language expert, fluent in Bengali and English, worked with the project leader to review the back-translation for discrepancies or inaccuracies. Upon confirmation of fidelity, the initial version underwent critical evaluation by an expert committee comprising two pain management clinicians, a language specialist, and the project leader. Their endorsement led to a second version that mirrored the original PCS precisely. Content validity was evaluated by the expert committee members using the item-level content validity index (I-CVI) and the scale-level content validity index (S-CVI). Content validity refers to the extent to which the items in the instrument are representative of the traditional theoretical construct that the instrument is designed to assess [31]. Each item was rated 1 (not relevant), 2 (somewhat relevant), 3 (quite relevant), or 4 (highly relevant). Ratings were dichotomized as relevant (3 or 4) and non-relevant (1 or 2). I-CVI was computed as the number of experts who rated an item as relevant divided by the total number of experts. The S-CVI was measured by averaging the I-CVIs for all items. S-CVIs of 1 and 0.9 were judged as excellent content validity, respectively [39]. A cognitive interviewer tested the translated items in this formatted version for comprehensibility, simplicity, appropriateness, and completion time. The patient sample size for cognitive debriefing was based on Perneger et al. (2015) [40]. Testing involved 10 male patients (mean age in years (SD): 57.70 (8.20)) and 20 female patients (mean age in years (SD): 53.2 (10.1)) with joint and low back pain lasting more than six months. Patients were literate and from rural and urban backgrounds, with minimal primary-level education. The interviewer recorded responses and any suggestions to improve the translated questionnaire. The project leader reviewed the interview results, which led to the production of the third version. A native Bengali speaker proficient in English proofread the third version. Following this step, the linguistically validated PCS for "Bengali for India" (BNGA-IND-PCS) was finalized and prepared for psychometric evaluation. The backward translation of the Bengali version of the Indian Pain Catastrophizing Questionnaire is provided in Appendix 1. The final Bengali version of the India Pain Catastrophizing Questionnaire is provided in Appendix 2. A flow diagram illustrating the entire process of linguistic validation and psychometric analysis is presented in Figure 1.

Assessment of psychometric properties

Exploratory factor analysis (EFA), confirmatory factor analysis (CFA), internal consistency, test-retest reliability, and concurrent validity were performed using the IBM SPSS Statistics version 27.0.1 (IBM Corp., Armonk, NY). Exploratory principal component analysis (PCA) was performed to maintain consistency with the original PCS [7] using the varimax rotation method and Kaiser normalization (Eigenvalues > 1).

CFA was conducted using LISREL 8.80 (Scientific Software International, Chapel Hill, NC). Model fit was evaluated using the chi-square statistic, root mean square error of approximation (RMSEA), and comparative fit index (CFI). A lower chi-square value indicates a better fit. A CFI near one is considered an excellent fit. RMSEA < 0.06 is regarded as an excellent fit, and 0.06 < RMSEA < 0.08 is considered satisfactory [35]. The Akaike Information Criterion (AIC) was used to assess whether a higher-order model potentially overfits the data. Among competing models, the one with the lower AIC is typically preferred. The internal consistency, indexed by Cronbach’s α, represents the degree of interrelatedness among the translated items. Instruments with a Cronbach’s alpha > 0.7 are considered adequate [9,35].

Retests for 100 patients from the hospital and clinic were self-administered three weeks after the baseline recording to assess test-retest reliability. Patients did not receive any change in treatment or intervention between the test and retest time points. Enrolled patients who had not subjectively perceived any change in their pain problem (worsening or improvement) three weeks after their previous visit were considered stable and included in the test-retest reliability analysis. Results were expressed using the intraclass correlation coefficient (ICC). An ICC ≥ 0.70 is considered indicative of acceptable reliability. Other recommended parameters, including the standard error of measurement (SEM) and minimal detectable change (MDC), were also calculated to assess reliability. A lower SEM indicates less variability. SEM was calculated using SEM = SD change × √(1 - ICC), where SD change is the change in the standard deviation of BNGA-IND-PCS scores between two time points. The 95% confidence interval of MDC (MDC95%) of the BNGA-IND-PCS was calculated using MDC95% = 1.96 × √2 × SEM. Limits of agreement with a 95% confidence interval (LOA95%) were computed from the mean difference ± 1.96 × SD change. A Bland-Altman plot was created to illustrate the limits of agreement for the total score.

Concurrent validity was assessed using the Spearman rank coefficient (ρ) between subscales and total scores of BNGA-IND-PCS, with scores of the Numerical Pain Rating Scale (NPRS) [23], Bengali translated and English version of the Patient Health Questionnaire (PHQ-9) [24,25], and Generalized Anxiety Disorder-7 (GAD-7) [41,42]. PHQ-9 is a brief, valid, and reliable measure of depression severity. GAD-7 was developed to assess anxiety in primary care settings. Usually, ρ > 0.8 is considered excellent [9,35]. The Shapiro-Wilk test was used to assess the normality of the data.

## Results

Translation acceptability

The translation was done in colloquial Bengali to make it easier for people with lower levels of education to understand. No problems were reported with any items in the BNGA-IND-PCS during the cognitive interview. All items were straightforward for patients to interpret without the interviewer's help. The average (SD) time to complete the BNGA-IND-PCS was 5.2 (1.54) minutes. Among completers, there was no missing data on item ratings for the scale. All items on the scale demonstrated excellent content validity, with I-CVI and S-CVI values of 1.

Demographics

Total BNGA-IND-PCS and subscales were normally distributed. The mean (SD) total BNGA-IND-PCS score was 24.5 (11.9). Demographic details of the patients and the descriptive statistics are provided in Table 1.

**Table 1:** Sociodemographic and clinical characteristics of the patients. CLBP: chronic low back pain; LBP: low back pain; MSD: musculoskeletal disorders. ^*^ Pain intensity was evaluated using the Numerical Pain Rating Scale (NPRS). ^#^ Depression and anxiety were assessed using the Patient Health Questionnaire (PHQ-9) and the Generalized Anxiety Disorder 7-item scale (GAD-7).

Variable	Value
Demographics	
Total number of patients, n (%)	253 (100)
Age in years (Mean ± SD)	54.7 ± 12.62
Gender distribution, n (%)	
Female	153 (60.47)
Male	100 (39.53)
Diverse chronic pain, n (%)	
Chronic low back pain (CLBP)	52 (20.55)
LBP + osteoarthritis of the knee joint	35 (13.83)
Lumbar radiculopathy	22 (8.70)
Osteoarthrosis	78 (30.83)
Musculoskeletal disorders (MSD)	44 (17.39)
Rheumatoid arthritis	22 (8.70)
Pain duration, n (%)	
6 months-1 year	123 (48.62)
2-3 years	48 (18.97)
4-5 years	34 (13.44)
>5 years	48 (18.97)
NPRS^*^ (0-10) (Mean ± SD)	6.82 (1.80)
Pain intensity distribution - NPRS^*^, n (%)	
Mild (0-4)	26 (10.28)
Moderate (5-6)	88 (34.78)
Severe (7-10)	139 (54.94)
Residential distribution, n (%)	
Rural	112 (44.27)
Urban	141 (55.73)
Educational level (% of patients), n (%)	
Below 5th grade (< 5th grade)	58 (22.92)
5th grade and above (≥ 5th grade)	195 (77.08)
Other outcome measures (Mean ± SD)	
PHQ-9^#^	7.95 ± 5.05
GAD-7^#^	7.04 ± 4.53

Psychometric validation

Exploratory Factor Analysis

Principal component analysis extracted a two-factor structure model that collectively explained 59.5% of the total variance. The first factor explained 51.7% of the total variance and included items 2, 3, 7, and 9-13, with factor loadings ranging from 0.490 to 0.784 (Table 2).

**Table 2:** BNGA-IND-PCS factor structure obtained using the principal component analysis. BNGA-IND-PCS: Bengali translation of the Pain Catastrophizing Scale developed in India. Extraction method: Principal component analysis using Varimax rotation and Kaiser normalization. Rotation converged in three iterations. The model explained 59.5% of the variance. Bold items represent the loadings for factors 1 and 2.

Item No.	Original subscale	Brief description	Factor 1	Factor 2
12	Helplessness	Nothing I can do	0.784	0.121
13	Magnification	Something serious may happen	0.731	0.259
3	Helplessness	Never get any better	0.686	0.276
7	Magnification	Thinking of painful experiences	0.640	0.238
9	Rumination	Can’t keep out of mind	0.631	0.502
2	Helplessness	Can’t go on	0.595	0.407
10	Rumination	Thinking how much it hurts	0.588	0.577
11	Rumination	Want the pain to stop	0.490	0.427
4	Helplessness	It’s awful	0.140	0.850
5	Helplessness	Can’t stand it	0.213	0.771
8	Rumination	Want the pain to go away	0.392	0.692
1	Helplessness	Whether pain will end	0.391	0.655
6	Magnification	Afraid if pain may get worse	0.547	0.573

Extraction method: Principal component analysis using Varimax rotation and Kaiser normalization. Rotation converged in three iterations. The model explained 59.5% of the variance. The item retention threshold was set above 0.45. Bold items represent the loadings for factors 1 and 2. The scree plot showed two components that were eligible according to Kaiser criteria (as provided in the Appendices).

The second factor explained 7.8% of the total variance and included items 1, 4-6, and 8 with factor loadings ranging from 0.573 to 0.850. Three rumination items (items 9-11), three helplessness items (items 2, 3, 12), and two magnification items (items 7 and 13) from the original scale were identified in factor 1. Factor 1 of the BNGA-IND-PCS appeared to indicate a state of repetitive focus on pain and was named "pain reflection." Three helplessness items and one item each from the rumination and magnification subscales of the original PCS were merged into factor 2. Factor 2 appeared to indicate an inability to cope. Factor 2 was named "helplessness."

The mean (SD) pain reflection and helplessness scores were 13.82 (7.790) and 10.69 (4.813), respectively. Significantly strong correlations were observed between the total score of the BNGA-IND-PCS and pain reflection (ρ = 0.964; P < 0.001), BNGA-IND-PCS and helplessness (ρ = 0.911; P < 0.001), and helplessness with pain reflection (ρ = 0.773; P < 0.001).

Confirmatory Factor Analysis

The goodness-of-fit indices (chi-square, RMSEA, and CFI) indicated that the two-factor model, derived from the EFA, provided the best fit compared with Sullivan’s three-factor model [7], Osman’s two-factor model [14], and the one-factor model (Table 3).

**Table 3:** Goodness-of-fit values and AIC for the different models tested. AIC: Akaike Information Criterion; CFI: comparative fit index; RMSEA: root mean square error of approximation. Information criteria, n = 253. Model 1: All items in a single-factor model. Model 2: Factor structure suggested by Osman et al. (1997) [14]. Model 3: Factor structure suggested in the original Pain Catastrophizing Scale by Sullivan et al. (1995) [7]. Model 4: Two-factor structure found in the present study.

		Chi-square (χ^2^)	df	χ^2^/df	RMSEA	CFI	Model AIC
Model 1	One factor (13 items)	150.42	65	2.31	0.07	0.98	202.42
Model 2	Two oblique factors (7+6 items)	146.73	64	2.29	0.07	0.98	200.73
Model 3	Three oblique factors (6+3+4 items)	144.61	62	2.33	0.07	0.98	202.61
Model 4	Two varimax components (8+5 items)	123.32	64	1.92	0.06	0.99	177.32

**Figure 2:**
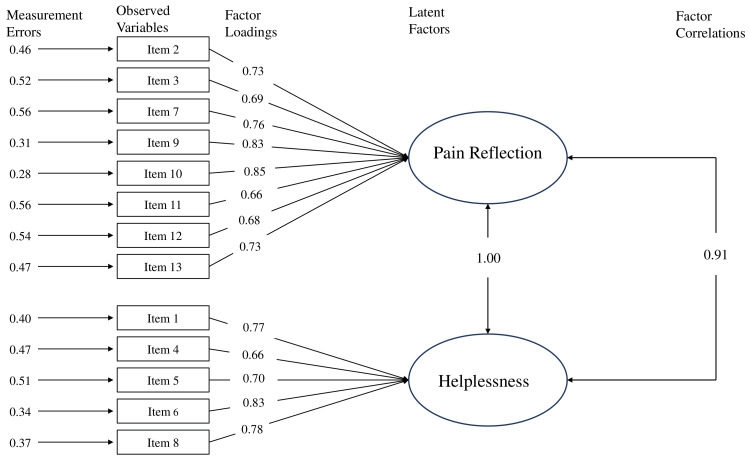
Confirmatory factor analysis (CFA) path diagram of a two-factor model of the BNGA-IND-PCS with adjustment for covariance of error terms (n = 253). BNGA-IND-PCS: Bengali translation of the Pain Catastrophizing Scale developed in India.

The lowest AIC value for the two-factor model suggests minimal risk of overfitting compared to others. The path diagram of the CFA model fit is included in Figure 2.

Internal Consistency

Cronbach’s α values for the internal consistency of pain reflection, helplessness, and total BNGA-IND-PCS scores were 0.88, 0.854, and 0.921, respectively (Table 4). All α values remained below the total BNGA-IND-PCS Cronbach’s α when any single item was removed. The item-total correlation coefficients ranged from 0.565 to 0.741.

**Table 4:** Cronbach’s α, alpha if item deleted, and item-total correlations. BNGA-IND-PCS: Bengali translation of the Pain Catastrophizing Scale developed in India. Information criteria, n = 253.

	α	Alpha (if item deleted)	Corrected item-total correlation
Pain reflection​	0.880		
Item 2		0.866	0.640
Item 3		0.868	0.625
Item 7		0.873	0.565
Item 9		0.857	0.730
Item 10		0.856	0.741
Item 11		0.873	0.570
Item 12		0.867	0.638
Item 13		0.865	0.655
Helplessness	0.854		
Item 1		0.826	0.667
Item 4		0.822	0.681
Item 5		0.832	0.634
Item 6		0.824	0.668
Item 8		0.816	0.698
Total BNGA-IND-PCS	0.921		

Test-Retest Reliability

**Figure 3:**
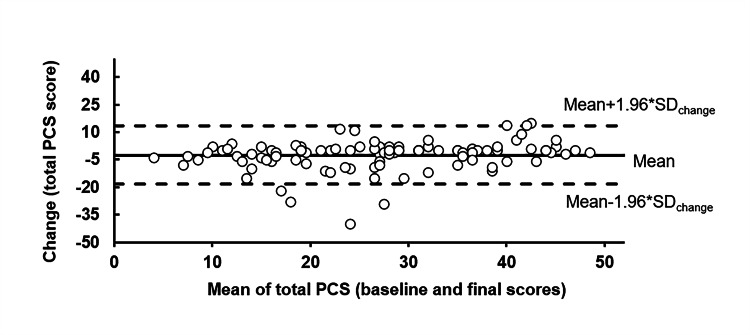
Bland-Altman plot for total PCS scores in the BNGA-IND-PCS. The y-axis denotes the change in the BNGA-IND-PCS total scores between baseline and follow-up measurements. The x-axis denotes the mean of the BNGA-IND-PCS scores at the baseline and final measurements. The black solid line is the mean change of score (mean), and the top and bottom dotted lines are mean ± Z x SD change (where Z = 1.96 for 95% confidence interval). PCS: Pain Catastrophizing Scale; BNGA-IND-PCS: Bengali translation of the Pain Catastrophizing Scale developed in India.

The test-retest reliability of BNGA-IND-PCS scores ranged from 0.830 to 0.879 (Table 5). Specifically, the ICCs (95% CI) for the pain reflection and helplessness subscales were 0.830 (0.733-0.889) and 0.879 (0.821-0.919), respectively. The ICC for the total BNGA-IND-PCS score was 0.852 (0.772-0.903). The SEM and MDC for the total BNGA-IND-PCS scores were 3.14 and 8.70, respectively. The 95% limits of agreement (LOA) ranged from -18.37 to 13.49 for the total PCS scores. A Bland-Altman plot illustrating the limits of agreement for the total BNGA-IND-PCS items is provided in Figure 3.

**Table 5:** Test-retest reliability. BNGA-IND-PCS: Bengali translation of the Pain Catastrophizing Scale developed in India; CI: confidence interval; ICC: intraclass correlation coefficient; LOA95%: limits of agreement at 95% confidence level; MDC95%: minimum detectable change at 95% confidence level; SEM: standard error of measurement. Information criteria, n = 100.

	SEM	MDC_95%_	LOA_95%_	ICC (95% CI)	P-value of ICC
Pain reflection	2.19	6.07	-12.95 to 9.23	0.830 (0.733-0.889)	<0.01
Helplessness	1.09	3.02	-6.30 to 5.52	0.879 (0.821-0.919)	<0.01
Total BNGA-IND-PCS score	3.14	8.70	-18.37 to 13.49	0.852 (0.772-0.903)	<0.01

Validity

To test the a priori hypothesis that PC is positively associated with pain severity, depression, and anxiety, concurrent validity analyses showed that total BNGA-IND-PCS scores were significantly and moderately correlated with NPRS (ρ = 0.483; P < 0.001), PHQ-9 (ρ = 0.439; P < 0.001), and GAD-7 (ρ = 0.461; P < 0.001). The correlations between pain reflection and NPRS (ρ = 0.412; P < 0.001), PHQ-9 (ρ = 0.406; P < 0.001), and GAD-7 (ρ = 0.441; P < 0.001) were significant. Significant correlations were also present between the helplessness and NPRS (ρ = 0.549; P < 0.001), PHQ-9 (ρ = 0.427; P < 0.001), and GAD-7 (ρ = 0.425; P < 0.001).

## Discussion

This study developed a reliable BNGA-IND-PCS version for assessing PC in patients with chronic musculoskeletal pain. After cultural adaptation, each item from the original PCS was retained, making the BNGA-IND-PCS version easy to self-administer. The structural validity, internal consistency, test-retest reliability, and concurrent validity of the BNGA-IND-PCS were within the acceptable limits of the COSMIN criteria.

The present study emphasized a Bengali PCS version that would be culturally appropriate for Bengali-speaking Indian patients, based on a large sample of native Bengali patients from multiple pain care settings. It examined the factor structure of the Bengali-adapted version of the PCS for the first time. These steps differed from those used in a previous Bangladeshi Bengali version of the PCS [31]. In contrast, the Bangladeshi PCS version assessed the content validity of all translated items from the original PCS but did not examine the factor structure [31]. This study maintained the conceptual equivalence of each original item's feelings and thoughts, which were confirmed by the items' relevance, comprehensiveness, and comprehensibility (content validity) for the specific population. The present study further ensured the subdomains of PC using EFA and verified them with CFA [35]. Hence, this study represents the first cross-cultural adaptation of a Bengali version of the original PCS in the Indian subcontinent, using EFA and CFA to verify the subdomains of the theoretical pain appraisal model critically.

Previous cross-culturally adapted versions of the PCS have reported either a three-factor [9] or a two-factor structure [9,14,16,19]. The BNGA-IND-PCS has been found to exhibit a two-factor structure with relatively low variance by the second factor. However, this was consistent with the previous two-factor models that accounted for 7.7% to 8.5% of the total variance [14,15]. Previously, the Nepalese version of the PCS in the Indian subcontinent had exhibited a two-factor solution, in which the first factor primarily included items related to helplessness, and the second factor comprised items related to magnification and rumination, which were associated with attention to pain [19]. The two-factor solution was first reported during testing of the original PCS with participants from North America [14]. The two-factor structure was also supported in a study involving African American and Caucasian workers with low back injuries [43] and in the Arabic PCS version [16].

Item-factor loadings may vary across cross-culturally adapted versions, reflecting how different cultures influence catastrophizing thoughts. In BNGA-IND-PCS, factors 1 and 2 each include three items related to helplessness. Factor 1 also includes three items related to rumination and two to magnification, whereas factor 2 includes one rumination and one magnification item. The items comprising factor 1 indicate heightened worry and repetitive, pain-related, intrusive thoughts of threat. Original helplessness items 2, 3, and 12 in this specific clinical population from Bengali culture reflect intense negative appraisal rather than an inability to cope. Hence, factor 1 appears to be associated with pain focus or pain reflection, while factor 2 exhibits perturbed coping ability or helplessness in patients with ongoing pain. Our preliminary validation aligns with the two-factor model rather than the traditional three-factor model. Previous literature on two-factor PCS has mainly treated helplessness as an independent factor and combined the rumination and magnification items into a single factor [14,19]. Usually, these subscales are named helplessness and pain focus or pain reflection, based on the theoretical foundation of impaired pain-coping ability and repetitive, negative, intrusive thoughts related to pain. Our exploratory analysis revealed that factor 2 resembles helplessness, and factor 1 reflects pain reflection. Notably, despite keeping the item retention threshold above 0.45, items 6, 9, and 10 showed cross-loadings. Two rumination items, "I can’t seem to keep it out of my mind" and "I keep thinking about how much it hurts," also exhibited out-of-control pain in approximately 90% of patients experiencing moderate to intense pain (NPRS scores) and hence secondary loading in factor 1. Similarly, the magnification item, "I become afraid that the pain will get worse," also exhibited clinical entanglement with worry. Secondary loading has been a concern even in some items in the original PCS, Osman’s two-factor, and the Nepalese version of PCS [14,19]. This might be due to the interrelatedness of factors reflecting semantic overlap across those cultures, rather than to poor item translation. We retained cross-loaded items due to their high content validity and assigned them to the factor with the higher loading. At this point, it is premature to decide on the deletion of these items until further verification is performed in a broader, more generalized Bengali population. The goodness-of-fit parameters showed that the BNGA-IND-PCS demonstrated the best model fit compared to Sullivan’s original three-factor model [7], Osman’s two-factor model [14], and the single-factor model. AIC differences of less than 2 are considered negligible, while differences above 10 are substantial. The gap between model 4 and the next-best Osman’s two-factor model (model 2, AIC = 200.73) was large, indicating item drift and likely a difference in the target population's conceptualization of the constructs from that tested in a culturally different population [14]. The RMSEA of 0.06 and the CFI of 0.99 indicated a good model fit for the BNGA-IND-PCS. However, our results could not be compared to the two-factor Nepalese and Arabic versions as they were tested on independent samples [16,19]. AIC also showed that the risk of overfitting was lowest in the BNGA-IND-PCS model compared with other models. Internal consistency, measuring the degree of interrelatedness among items, demonstrated a high Cronbach’s α value (α = 0.921) in the BNGA-IND-PCS total score, meeting the excellent standard set by COSMIN criteria. Internal consistency was similar to that of the total PCS score in the Bangladeshi version [31] and to several cross-culturally adapted and translated PCS versions [9]. The high Cronbach’s α value above 0.9, indicating excellent reliability, could also suggest possible item redundancy. Item-level redundancy in PCS arises during cross-cultural adaptation due to closely related concepts that cause semantic overlap and mutually correlated subcomponents [44]. The subscales of pain reflection in factor 1 and factor 2 also exhibited high Cronbach’s α values comparable to those found in the Nepalese version [19].

The test-retest reliability of chronic PC (total BNGA-IND-PCS), assessed in 100 patients twice over a three-week interval, was excellent, with an ICC above 0.8. Helplessness and pain reflection also showed excellent reliability. The ICC for the total PCS score was higher than that of the Bengali version from Bangladesh, tested at a 14-day interval [31]. This interval was carefully selected to avoid both memory bias and fatigue from repeated administration at shorter intervals [35]. Previous studies assessing test-retest reliability over three to four weeks have also shown good to excellent reliability in relatively small patient samples [28,45]. We calculated the SEM using the recommended COSMIN guidelines to assess the precision of within-sample scores based on reliability estimates [20]. Instruments with larger SEMs are less precise, since a small change in the actual score cannot be isolated from systematic and random error. Conversely, lower SEM values indicate greater measurement precision. Our SEM for the total PCS score (3.14) remained within the reported thresholds (1.6 to 4.6) from previous PCS literature [18-20,28,46,47]. SEM values were lower in the Hindi [20] and Nepalese [19] versions, possibly due to comparatively shorter retest intervals. To further validate the clinically meaningful stability of our version, the MDC for the total PCS score was estimated at 8.7. Thus, a total PCS score change of 9 in BNGA-IND-PCS can be considered indicative of a true change beyond measurement error. This value was slightly below the range of 8.8-13 observed in other validation studies [27,28,46,47]. Our MDC reportedly falls within the minimal detectable change thresholds of 8 and 11 for low and high catastrophizers, respectively [27]. The Bland-Altman plots examined the extent to which total PCS scores deviated from the mean change in responses between the test and retest administrations. The mean bias of -2.44 for the total PCS score indicated that participants, on average, scored slightly lower during the retest. It is speculated that the significant reduction in a patient's PCS score might be due to desensitization from repeated administration [48]. Visual inspection of the plot indicated consistency between the measurements. The plot exhibited a line of agreement (LOA) between test and retest scores, suggesting that 95% of patients’ follow-up assessments would be between 18.37 points less and 13.49 points greater than the baseline measurement. Despite sufficient to acceptable test-retest stability measures, the LOAs were large. This is a very wide range relative to the scale's dispersion, suggesting substantial individual-level variability between test and retest occasions that the ICC alone (0.852) partially obscures. The large LOA observed in the plot may reflect that these patients with unaltered moderate-to-severe pain at retest had diverse educational backgrounds and musculoskeletal conditions. Moreover, we caution that the LOA should be reinvestigated at shorter test-retest intervals before the BNGA-IND-PCS is used in research and clinical settings.

PC shares some analogous elements of attentional and processing bias (a negative cognitive-affective construct) associated with anxiety and depression in chronic pain [10]. Hence, anxiety and depression are common mental health issues in chronic pain, contributing to adverse health outcomes [8,9]. Previous studies on PCS adaptation have used anxiety and depression measures to examine their associations with PC as part of concurrent validity assessments [9,27,49]. As expected, a significant moderate positive correlation with PC was found. Significant moderate correlations between the BNGA-IND-PCS scores and the scores of pain severity, depression, and anxiety were recorded within the previously reported range [19,27,47,49-51]. The PHQ-9 and GAD-7 scores indicated mild depression and anxiety symptoms in patients and were below the cut-off level.

Strengths and limitations

The multicenter design and sample size, comprising rural and urban patients with chronic pain, were strengths of the present study. One methodological strength was a rigorous translation and linguistic validation process conducted in accordance with recognized international guidelines, involving blinded back-translation, content validity estimation by expert panels, and cognitive interviewing with a reasonably sized pilot group. Another rigorous approach was using CFA following EFA, rather than performing CFA alone with an assumed structure. This was the core strength that distinguishes our study from the Bangladesh PCS version, which lacked a factor-analytic evaluation of latent pain catastrophizing factors in the Bengali population. The study findings met the a priori hypothesis in accordance with the recommended COSMIN guidelines. Adherence to the COSMIN framework for psychometric evaluation provided transparency, enabling direct comparison with other PCS adaptations, which was also a strength of the work. However, there were several limitations of the study. The primary limitation was the lack of an independent validation sample. Both EFA and CFA were conducted on the same sample, a methodological limitation that resulted in circular validation. Although the factor structure has been demonstrated using CFA as a model-comparison exercise in the same sample, the study lacked independent confirmatory evidence of structural validity at this point. The use of exploratory PCA rather than EFA was another limitation, as the latter is considered a reliable construct validation procedure. However, we chose PCA to maintain consistency with the method used in the developer's original PCS. Assessment of measurement invariance across subgroups (e.g., gender, age, demographic distribution, enrollment categories, literacy levels, and six different musculoskeletal conditions) was not possible due to insufficient power. These heterogeneities could be potential confounds limiting the precision of the factor solution. The instrument was intended for the study's target population. However, this potential selection bias excluded the general population from normative validation, which was a limitation of the study. To minimize fatigue and time demands for patients experiencing pain, we opted not to include additional scales to assess discriminant validity. However, because the concurrent validity with the PHQ-9 and GAD-7 was moderate, demonstrating discriminant validity would have been important; we acknowledged this as a limitation in this study. Another limitation of the study was its single-sample design, which introduced regional bias and limited the generalizability of the results. Another limitation was the lack of longitudinal follow-up of changes in PC sensitivity in the population under long-term treatment, which could have further increased the versatility of the BNGA-IND-PCS.

## Conclusions

In conclusion, the preliminary validation of the Bengali version of the PCS demonstrated acceptable psychometric performance in this patient population. A two-factor solution showed the best fit in this single-sample study, though it is premature to claim it is definitive until independently validated. The clinical utility in lower-severity or non-treatment-seeking populations has not been established. Hence, to ensure the "ecological validity" of the BNGA-IND-PCS, future steps are needed to clarify how catastrophizing shapes the chronic pain experience in the wider Bengali population. Future work should include multi-sample validation of the factor structure, invariance testing across demographic groups, and prospective studies using independent cohorts.

## Figures and Tables

**Figure 4:**
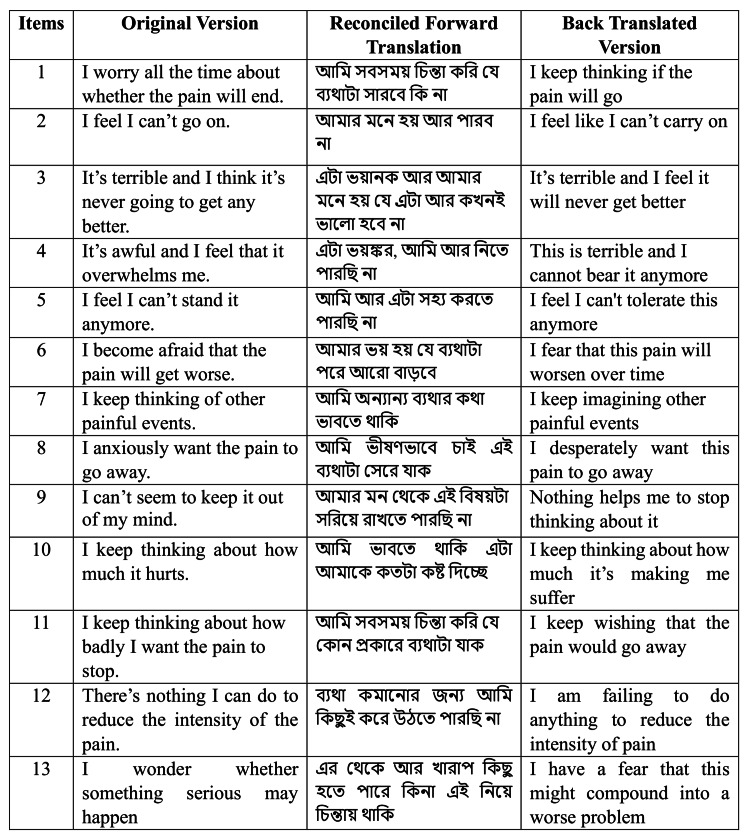
Backward translation of the Bengali Pain Catastrophizing Scale developed in India.

**Figure 5:**
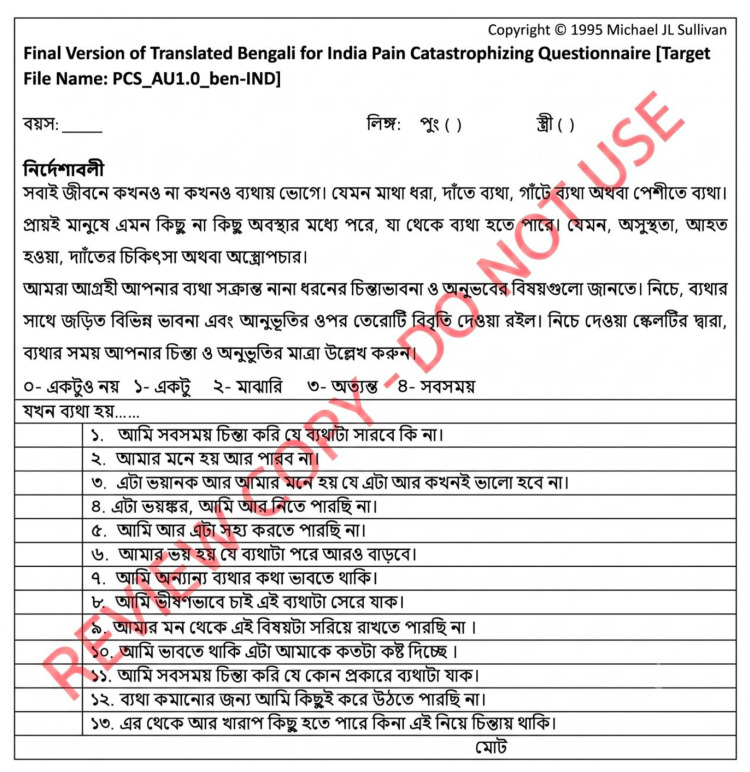
The final Bengali version of the Pain Catastrophizing Scale (PCS) for India. PCS Licensing managed by MRT. Permission to use: Mapi Research Trust, Lyon, France, https://eprovide.mapi-trust.org Official PCS | Pain Catastrophizing Scale distributed by Mapi Research Trust | ePROVIDE: https://eprovide.mapi-trust.org/instruments/pain-catastrophizing-scale

**Figure 6:**
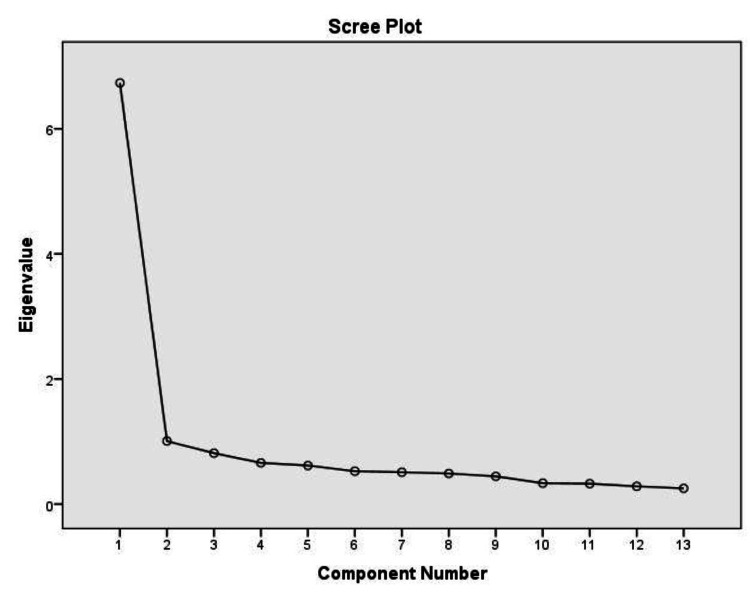
Scree plot of principal component analysis for the Bengali Version of the Pain Catastrophizing Scale (BNGA-IND-PCS). Scree plot from principal component analysis (Varimax rotation) of the 13-item Bengali version of the Pain Catastrophizing Scale (BNGA-IND-PCS) in musculoskeletal chronic pain patients. Two components demonstrated eigenvalues exceeding 1.0 per the Kaiser criterion.

## Appendices

Appendix 1

Appendix 2

Scree plot
