# Correction: Structure, Reliability, and Validity of the Bengali Version of the Pain Catastrophizing Scale in Musculoskeletal Chronic Pain Patients in India

**DOI:** 10.7759/cureus.c464

**Published:** 2026-07-20

**Authors:** Soumyajyoti Ghoshal, Santi Ranjan Dasgupta, Subhadip Paul, Arkadeb Dutta

**Affiliations:** 1 Department of Biomedical Science and Technology, Ramakrishna Mission Vivekananda Educational and Research Institute (RKMVERI), Kolkata, IND; 2 Department of Orthopaedics and Pain Management, ESI (Employees' State Insurance) Institute of Pain Management, Kolkata, IND; 3 Department of Sports Science and Yoga, Ramakrishna Mission Vivekananda Educational and Research Institute (RKMVERI), Kolkata, IND

This article has been corrected to replace Figure 5 in the appendices with a watermarked version. Licensing information has also been added to the figure legend.

